# Ramucirumab: A New Therapy for Advanced Gastric Cancer

**DOI:** 10.6004/jadpro.2015.6.1.8

**Published:** 2015-01-01

**Authors:** Andrea Landgraf Oholendt, Jennifer L. Zadlo

**Affiliations:** The University of Texas MD Anderson Cancer Center, Houston, Texas

Gastric cancer is the fourth most common cancer in men and the fifth most common cancer in women worldwide (Jemal et al., 2011). The American Cancer Society (ACS) has estimated there were 22,220 new cases diagnosed in 2014, with an estimated 10,990 deaths (Siegel, Ma, Zou, & Jemal, 2014). Despite some advances in treatment, gastric cancer continues to be associated with poor outcomes.

Approximately two-thirds of patients diagnosed with gastric cancer have locally advanced or metastatic disease, with a median survival of around 10 months (Chau et al., 2004). Chemotherapy is the primary modality for management of advanced and metastatic gastric cancers and has been shown to improve quality of life and prolong survival (Wagner et al., 2006). The standard of care for treatment of advanced disease is poorly defined, with no preferred first- or second-line regimens.

Category 1 recommendations for first-line therapy for locally advanced and metastatic disease include a two-drug regimen of cisplatin combined with a fluoropyrimidine (fluorouracil or capecitabine) or cisplatin and a fluoropyrimidine combined with a third agent, which is typically epirubicin or docetaxel. Two-drug regimens are generally preferred, since they are associated with less toxicity (National Comprehensive Cancer Network [NCCN], 2014).

Prior to 2014, there were no category 1 recommendations for second-line therapy for gastric cancer. The decision to give second-line therapy was generally based on the patient’s performance status and prior therapy exposure. Preferred regimens included single-agent irinotecan or taxane (docetaxel or paclitaxel; NCCN, 2014). The development of new therapies offers viable treatment options for patients with advanced and metastatic disease.

## ANGIOGENESIS

Angiogenesis is an important process in cancer development and growth. Angiogenesis is primarily driven by the interactions between vascular endothelial growth factor (VEGF) ligands and VEGF receptors (VEGFRs). The VEGF ligands include VEGF-A, VEGF-B, VEGF-C, VEGF-D, VEGF-E, and placental growth factor (PGF; Claesson-Welsh & Welsh, 2013). Overexpression of VEGF has been associated with advanced-stage disease and poorer outcomes and prognosis in gastric cancer (Maeda et al., 1996).

Bevacizumab (Avastin) was the first antiangiogenic agent approved by the US Food and Drug Administration (FDA) and exerts its effect by preventing the binding of the VEGF-A ligand to its receptors. However, a phase III clinical trial did not demonstrate a significant improvement in overall survival when adding bevacizumab to chemotherapy for first-line treatment of advanced gastric cancer (Ohtsu et al., 2011).

Ramucirumab (Cyramza) is the first FDA-approved agent to reduce tumor angiogenesis by targeting the extracellular domain of the VEGFR. It remains unclear why ramucirumab has demonstrated efficacy for advanced gastric cancer while bevacizumab demonstrated mixed results. One rationale is ramucirumab blocks VEGFR-2, the primary receptor for VEGF-A, rather than blocking the ligand itself (Park, Thomas, Yoon, & Yoon, 2015; Spratlin, Mulder, & Mackey, 2010).

## MECHANISM OF ACTION

Ramucirumab is a recombinant human immunoglobulin G1 monoclonal antibody that binds to the extracellular binding domain of VEGFR-2 and prevents the binding of VEGFR ligands: VEGF-A, VEGF-C, and VEGF-D (Eli Lilly and Company, 2014; Wadhwa, Taketa, Sudo, Blum-Murphy, & Ajani, 2013). Through blocking activation of VEGFR-2 by VEGF-A and the other VEGF ligands, ramucirumab inhibits the angiogenesis pathways involved in the development and progression of gastric cancer.

## CLINICAL STUDIES

To date, there are two studies evaluating the efficacy of ramucirumab for the treatment of gastric and gastroesophageal (GE) junction adenocarcinoma. The REGARD trial was an international, prospective, double-blind, placebo-controlled, phase III study in adult patients with advanced or metastatic gastric or GE junction adenocarcinoma. Those patients who failed to respond to a first-line platinum or fluoropyrimidine-containing regimen were randomized in a 2:1 fashion to receive either ramucirumab plus best supportive care (BSC) or placebo plus BSC (Fuchs et al., 2014).

A total of 238 patients received ramucirumab intravenously (IV) at a dose of 8 mg/kg every 2 weeks, and 117 patients were randomized to the placebo arm. The study investigators found a statistically significant difference in the primary endpoint, median overall survival (OS). Patients who received ramucirumab plus BSC had a longer median OS, 5.2 months (range, 2.3–9.9 months), than did patients who received placebo plus BSC, 3.8 months (1.7–7.1 months); the hazard ratio was 0.776 (95% confidence interval [CI], 0.603–0.998), and the *p* value was .047. Of note, the longer median OS in the ramucirumab arm was preserved when repeated with multivariate analysis (*p* = .037; Fuchs et al., 2014).

Progression free survival (PFS) was also longer with ramucirumab plus BSC (2.1 months; range, 1.3–4.2 months) than with placebo plus BCS (1.3 months; range, 1.1–2.1 months); the hazard ratio was 0.483 (95% CI, 0.376–0.620), and the *p* value was < .0001. Also statistically significant was an increased disease control rate of 49% with ramucirumab plus BSC, compared with 23% with placebo plus BSC.

Best response was modest at best, typically stable disease (SD), which was seen in 45% of patients (n = 108) who received ramucirumab plus BSC, and 21% of patients who received placebo plus BSC (n = 24). Only one patient achieved a complete response, and seven patients (3%) achieved a partial response with ramucirumab plus BSC. Disease progressed in all others (Fuchs et al., 2014).

This study also included a 6-week quality-of-life assessment (QOL), to which data were available in 48% and 25% of the patients who received ramucirumab plus BSC and placebo plus BSC, respectively. Although not statistically significant (*p* = .23), there was a trend toward stable or improved global QOL reported in patients who received ramucirumab plus BSC. There was, however, a statistically significant longer time to deterioration in Eastern Cooperative Oncology Group (ECOG) performance status to a score of ≥ 2 in patients who received ramucirumab plus BSC (5.1 months) compared with placebo plus BSC (2.4 months; *p* = .002; Fuchs et al., 2014).

The RAINBOW trial was the second study to evaluate the efficacy of ramucirumab. The RAINBOW trial was an international, placebo-controlled, randomized, double-blind, phase III study of patients with advanced, nonresectable GE junction or gastric adenocarcinoma that had progressed within 4 months of receiving a first-line platinum and fluoropyrimidine-based chemotherapy regimen. Patients were randomized to receive ramucirumab at a dose of 8 mg/kg IV on days 1 and 15 plus paclitaxel 80 mg/m₂ IV on days 1, 8, and 15 of a 28-day cycle or placebo plus paclitaxel 80 mg/m₂ IV on days 1, 8, and 15 of a 28-day cycle; crossover was prohibited. A total of 665 patients were randomized; 330 patients received ramucirumab plus paclitaxel and 335 received placebo plus paclitaxel (Wilke et al., 2014).

This study found a statistically significant difference in the primary endpoint of median OS, which was longer in patients who received ramucirumab plus paclitaxel (9.6 months) than in those who received placebo plus paclitaxel (7.4 months; hazard ratio = 0.807; 95% CI, 0.678–0.962; *p* = .017). Improvements in median PFS were also observed with ramucirumab plus paclitaxel (4.4 months) vs. placebo plus paclitaxel (2.86 months; *p* < .0001). A majority of patients achieved SD: 52% in the ramucirumab plus paclitaxel arm vs. 47% in the paclitaxel plus placebo arm.

Partial response and complete response were achieved in 27% and < 1%, respectively, of those who received ramucirumab plus paclitaxel compared with 16% and < 1%, respectively, of those who received paclitaxel plus placebo. Of patients on ramucirumab plus paclitaxel and paclitaxel plus placebo, 13% and 25%, respectively, demonstrated progressive disease. The disease control rate was significantly higher in patients who received ramucirumab plus paclitaxel vs. paclitaxel plus placebo (80% vs. 64%, respectively; *p* < .0001; Wilke et al., 2014).

## SAFETY AND TOLERABILITY

The most common adverse drug reactions observed in patients who received ramucirumab monotherapy were hypertension, diarrhea, and bleeding/hemorrhage. Tables 1 and 2 highlight the relevant adverse drug reactions that were observed in the REGARD and RAINBOW trials (Fuchs et al., 2014; Wilke et al., 2014).

**Table 1:**
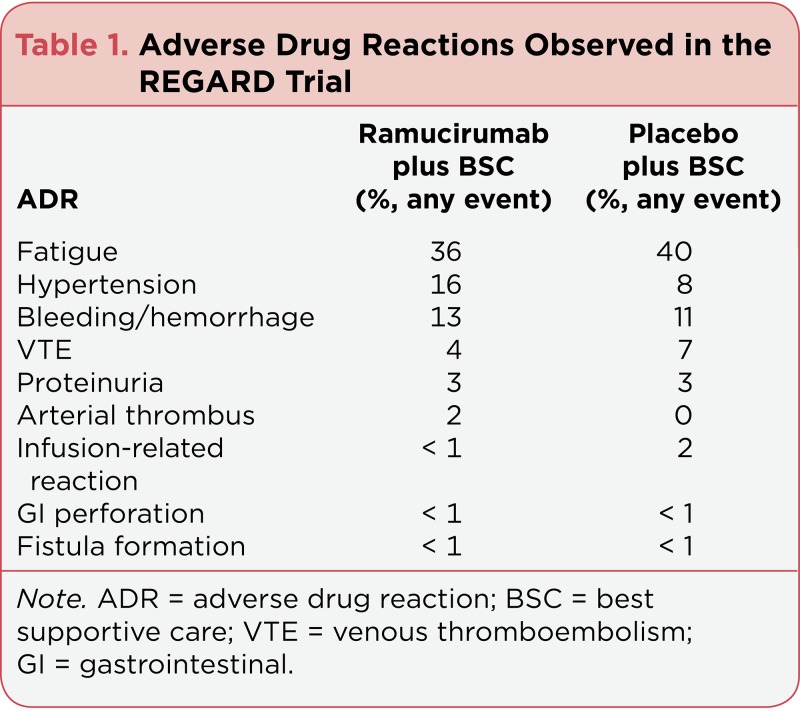
Adverse Drug Reactions Observed in the REGARD Trial

**Table 2:**
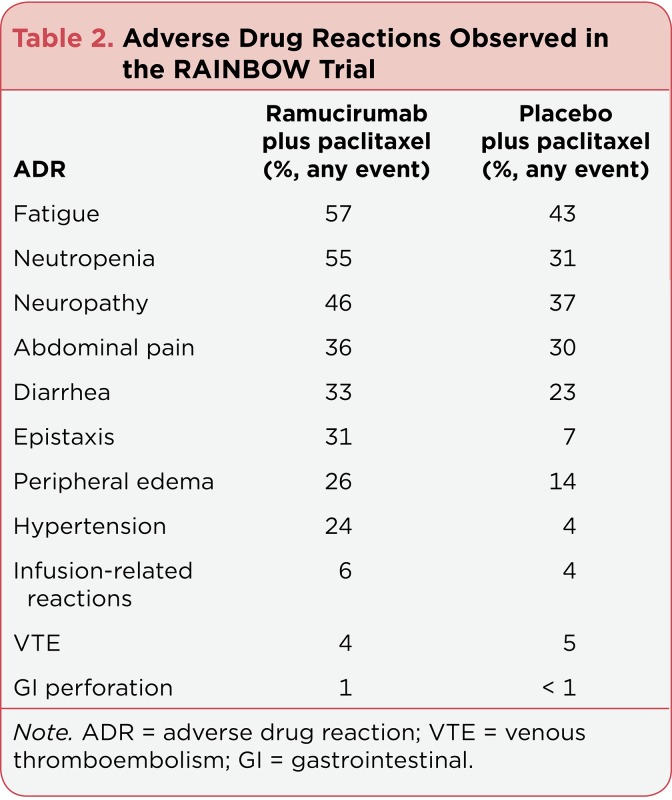
Adverse Drug Reactions Observed in the RAINBOW Trial

As demonstrated in Tables 1 and 2, the adverse drug reaction profile varied when ramucirumab was used as monotherapy vs. in combination with paclitaxel. Because of its antiangiogenic activity, concern for serious antiangiogenic adverse drug reactions, including GI perforations, impaired wound healing, venous and arterial thromboembolism, and hemorrhage, should be applied. Ramucirumab is currently labeled with a Boxed Warning for hemorrhage and serious bleeding events; however, the incidence of bleeding/hemorrhage was similar between ramucirumab and placebo in the REGARD trial.

Although ramucirumab is a recombinant human monoclonal antibody, infusion-related reactions have been reported in clinical studies. Therefore, premedication with an IV histamine (H1) antagonist is recommended by the manufacturer (Fuchs et al., 2014; Wilke et al., 2014; Eli Lilly and Company, 2014).

## DOSING

Based on the clinical data presented, ramucirumab is dosed at 8 mg/kg, using actual body weight, IV over 60 minutes, every 2 weeks. Currently, ramucirumab is approved by the FDA in the second-line setting for patients with gastric or GE junction adenocarcinoma, as monotherapy or in combination with paclitaxel, and continued until disease progression or unacceptable toxicity (Eli Lilly and Company, 2014).

## IMPLICATIONS FOR ITS USE IN CLINICAL PRACTICE

The FDA approval of ramucirumab has now introduced the use of biologic therapy into the treatment of gastric and GE junction adenocarcinomas. This step will require both clinicians and patients to become more familiar with this therapy and its unique toxicities.

Prior to ramucirumab administration, patients’ risk factors for bowel perforation/obstruction, bleeding/hemorrhage, and venous thromboembolism should be assessed. No specific risk factor assessment tools were used for assessing the risk of venous thromboembolism, perforation/obstruction, or bleeding complications in the REGARD or RAINBOW trial. However, clinicians should consider patient-specific risk factors and exercise best clinical judgment when evaluating a patient’s appropriateness for therapy. The inclusion/exclusion criteria for the REGARD and RAINBOW trials may be used as a reference for assessing a patient’s appropriateness for treatment; these criteria can be found in the respective indexes of the REGARD and RAINBOW studies.

Blood pressure should be controlled prior to the initiation of ramucirumab, monitored every 2 weeks, and continued to be well controlled throughout treatment. More specifically, the RAINBOW trial required a blood pressure goal of < 150/90 mm Hg for study inclusion. Patients should be educated on the signs and symptoms of venous thromboembolism, bleeding, home blood pressure monitoring, infusion-related reactions, and bowel perforation (Fuchs et al., 2014; Wilke et al., 2014; Eli Lilly and Company, 2014).

With the publication of the REGARD and RAINBOW trials, the FDA has approved ramucirumab, as a single agent or in combination with paclitaxel, as second-line therapy after failure of a fluoropyrimidine or platinum-containing chemotherapy regimen. The NCCN has already incorporated ramucirumab into its Guidelines for gastric cancer as a single agent or in combination with paclitaxel as a preferred second-line treatment (Eli Lilly and Company, 2014; NCCN, 2014).

Finally, it should be noted that the adverse drug reaction profile of ramucirumab differs when it is given as monotherapy vs. in combination with paclitaxel. Overall, a higher incidence of fatigue, neutropenia, diarrhea, epistaxis, and abdominal pain was reported in the RAINBOW trial compared with the REGARD trial. Attention to the adverse drug reaction profile should be remembered when considering the use of ramucirumab in clinical practice (Fuchs et al., 2014; Wilke et al., 2014; Eli Lilly and Company, 2014).

## SUMMARY

Treatment options for patients with locally advanced and metastatic gastric cancers are limited, and new therapies to improve QOL and prolong survival are needed. As the first monoclonal antibody to bind to the extracellular domain of the VEGFR-2, ramucirumab has demonstrated an improvement in progression-free survival and overall survival as monotherapy and in combination with paclitaxel in pretreated patients with advanced gastric cancer. With its manageable toxicity profile, ramucirumab provides practitioners with a safe and effective FDA-approved second-line therapy option for gastric cancer.
